# How Family Communication about Early-Stage Dementia is Silenced: Self-Censorship and Accommodation Strategies

**DOI:** 10.1093/geroni/igaf122.3604

**Published:** 2025-12-31

**Authors:** Shicheng Xu, Vivian Lou, Ernest Gonzales, Sehyun Baek

**Affiliations:** New York University, New York, New York, United States; The University of Hong Kong, Hong Kong, China (People’s Republic); New York University, New York, New York, United States; New York University, New York, New York, United States

## Abstract

The prevalence of Alzheimer’s disease and related dementias in China is significant, with an estimated 15.07 million people in 2020. However, this may be an underestimate as many older adults and family members avoid discussing dementia and undergoing assessment and treatment. This qualitative study explores intergenerational family communications styles on why it is difficult to broach dementia-related issues with family members, and how intergenerational family communication about early-stage dementia is silenced in Chinese families. Participants were recruited from Chinese social media and dementia care centers in Shanghai. Guided by constructivist grounded theory, we conducted three rounds (convenience sampling, theoretical sampling, member checking) of semi-structured interviews with 28 Chinese participants from three generations, including 9 people living with dementia (PLwD), 7 spouses of PLwD, 5 adult children of PLwD, and 6 adult grandchildren of PLwD. “Censoring communicability” emerged as a core category, with two properties: “conducting self-censorship” and “adjusting communication strategies”. There appears to be a tension between communication and self-censorship based on a continuum from communicating directly, informally, indirectly, matter-of-factly without emotions, and gradually lowering the level of communicability to silencing. Internalized hierarchy and designated roles within the family are the key barriers that reduced the opportunity for family members to communicate concerns about initial memory problems, help-seeking, and diagnostic disclosure, particularly with the family decision makers, who are usually the spouse or adult child caregivers. Direct and informal communication styles across three generations appear to be ideal pathways to seeking assessment and medical treatment.

